# Integrating immediate Kangaroo Mother Care as an integral part of small and sick newborn care in facilities with level 2 Special Newborn Care Units: Implementation Research Protocol

**DOI:** 10.12688/gatesopenres.16392.1

**Published:** 2026-07-03

**Authors:** iKMC-IR Study Group, Aarti Kumar, Ebunoluwa A Adejuyigbe, Abiy Seifu Estifanos, Ahmed Ehsanur Rahman, Abdullah H Baqui, Tarun Kumar, Pinky Jowel�, Henry C Anyabolu, Fitsum Woldegabriel Belay, Anisuddin Ahmed, Salahuddin Ahmed, Shrutika Dhiman, Ashok Kumar�, Ibrahim O Awowole, Mahlet Abayneh, Abu Sayeed, Mohammod Shahidullah, Bidhi Lord Singh, Shakal Narayan Singh, Tope Ojo, Mekdes Shifeta Argaw, Fariya Rahman, Ferdousi Begum, Shruti Bisht, Harish Chellani, Shuchita Gupta, Sachiyo Yoshida, Anayda Portela, Suman Rao, Anjoo Agarwal�, Akalewold Alemayehu, Abdul Mannan, Sabbir Ahmed, V K Anand, Vishwajeet Kumar, Tolulope Ogundele, Zerai Kassaye Tarrekegn, Shams El Arifeen, Kayur Mehta, Sarmila Mazumder, Sachiyo Yoshida, Anayda Portela

**Affiliations:** 1 World Health Organization, Geneva, Switzerland

**Keywords:** Immediate Kangaroo Mother Care, iKMC, low birthweight, preterm birth, mother-special newborn care unit (M-SNCU), skin-to-skin contact, breastfeeding, implementation research

## Abstract

**Background:**

Immediate Kangaroo Mother Care (iKMC) involves the initiation of skin-to-skin contact (SSC) between the mother (or an additional caregiver) and preterm and/or LBW infants within the first hours of birth, with exclusive breastfeeding. While KMC has traditionally been initiated after the infant stabilizes, immediate initiation of SSC has proven more effective in reducing neonatal mortality. However, despite its proven benefits, the adoption of immediate KMC remains a challenge. This study aims to develop and test an implementation model for integrating immediate KMC into the routine care of small and sick newborns in level 2 Special Newborn Care Units (SNCUs) in Bangladesh, Ethiopia, India, and Nigeria.

**Methods:**

This multicountry implementation research study will develop and evaluate a scalable model for integrating iKMC into newborn care across six study sites in four countries. The study will involve developing and optimizing an implementation model using a mixed-methods design, combining both qualitative and quantitative approaches. Formative research will be conducted to identify barriers and facilitators to the effective implementation of iKMC, guiding the development of the initial model. The study will benefit from concurrent programme learning, using both qualitative insights and outcome measurements, to iteratively refine the model and ensure high-quality, widespread implementation of iKMC.

**Conclusion:**

The study will provide insights into the effective integration of iKMC into existing health systems, addressing key challenges in low-resource settings. Findings will inform scalable strategies for widespread implementation, contributing to global efforts to enhance newborn care and reduce neonatal mortality.

**Abbreviations**
CHW: community health workers
CI: confidence interval
CPAP: continuous positive airway pressure
CRF: case report form
CVT: character varying type
ENAP: every newborn action plan
iKMC: immediate kangaroo mother care
IR: implementation research
KMC: kangaroo mother care
LBW: low birthweight
LMIC: low- and middle-income country
M-SNCU
: mother-special newborn care unit
MS SQL: Microsoft structured query language
NICU: neonatal intensive care unit
RCT: randomised controlled trial
RR: relative risk
SBCU: special baby care unit
SCANU: Special Care Newborn Unit
SNCU: special newborn care unit
SSC: skin to skin contact
WHO: World Health Organization

## 1. Background

Annually about 23.4 million infants are born small for gestational age, and 13.4 million are born preterm. ^1^ These infants are at a heightened risk of death and developmental challenges, contributing to 80% of all neonatal deaths. ^2^^,^^3^ Kangaroo mother care (KMC), involving prolonged skin-to-skin contact (SSC) with the mother, with exclusive breastmilk feeding is an effective evidence-based intervention for enhancing the survival, health, and development of these vulnerable infants.

Until recently, the global recommendation was to initiate KMC in health facilities after the infant is clinically stable. ^4^ However, by the time preterm and/or LBW infants become clinically stable, half to two-thirds of early deaths would already have occurred, limiting KMC’s effectiveness in preventing these deaths. ^5^ The impact could be much larger if KMC were initiated as soon as possible after birth, as demonstrated by the WHO-coordinated trial, which led to a 25% reduction in neonatal mortality (95% CI) when KMC was started within 2 hours after birth irrespective of the clinical condition of the infant vs. when it was started when the newborn was clinically stable. ^6^ In November 2022, WHO also updated its guidelines recommending iKMC as a standard of care for all preterm and/or LBW infants based on the latest systematic review indicating that KMC initiated early (defined as within 24 hours of birth) reduces neonatal mortality by 23% (RR 0.77, CI 0.66 to 0.91), compared to late-initiated KMC. ^7^ In addition to early initiation, the duration of KMC is important. Greater mortality reduction was observed when the daily duration of KMC was at least 8 hours per day than with shorter-duration KMC. ^8^

However, starting KMC within a few hours after birth when the infant is very small or sick requires specialized/intensive care, and sustaining it for up to 24 hours per day requires medical interventions such as respiratory support (e.g., CPAP), intravenous fluids, and close physiological monitoring. Ensuring prolonged skin-to-skin contact while maintaining these medical treatments requires specialized infrastructure and equipment that can accommodate mothers alongside their infants, trained healthcare staff to support mothers in providing KMC while managing the infant’s clinical needs, and policies that enable zero separation. These elements are essential for sustaining iKMC for up to 24 hours daily.

Hence, integrating iKMC into existing health systems may pose significant challenges, particularly in resource-limited settings where healthcare infrastructure and health provider numbers and capacity may not be adequate. This becomes even more challenging when we consider that global coverage of KMC, even for stable preterm infants, remains very low despite 20 years of existing WHO recommendations and global impetus on improving its implementation. iKMC is a new concept that challenges the traditional model of care wherein mothers are separated from their very small and/or sick infants when they require admission to the newborn care unit, and the care is taken over primarily by the healthcare workers.

The new concept requires that the mother and infant remain together at all times, even when the infant is small or sick, a concept that is virtually non-existent even in high-income/well-resourced settings. It poses challenges into the mindset of health professionals who are traditionally trained to view the separation of mother and infant as necessary for specialised care in special newborn care units (SNCU), and who see the mother’s role as limited to that of a visitor rather than an active caregiver.

Although evidence on efficacy is clear, there is an evidence gap on how to effectively integrate iKMC into routine small and/or sick newborn care (SSNC). ^9^^–^^11^ These challenges necessitate implementation research to identify effective strategies for integrating iKMC as standard of care for small and/or sick infants and to address context-specific barriers.

This iKMC implementation research (iKMC-IR) study aims to develop a model to effectively integrate iKMC into the care of small and/or sick infants in the existing health systems and test the model for optimisation in level 2 facilities at 6 study sites in 4 countries.

This study will be conducted in two phases. Phase 1 will focus on the development and optimisation of an implementation model and phase 2 will focus on the scale up of the optimized implementation model in multiple districts in the study countries. This protocol paper outlines the aim, objectives, and study design of phase 1 of the iKMC-IR study.

## 2. Methods/Design

### 2.1 Objectives

1. In phase 1, to develop an optimized implementation model in six sites in four countries (Bangladesh, Ethiopia, India, and Nigeria) that will add iKMC to functional systems of care for preterm or LBW infants. This model will provide necessary care including iKMC (skin-to-skin contact and breastmilk feeding), respiratory support, warmth, monitoring, and prevention and treatment of infections in newborn care units. The model will transform level 2 NICUs to level 2 M-SNCUs in health administrative areas (e.g., 1-2 districts) in four countries.
2. In phase 2, to demonstrate the scalability of the optimized iKMC implementation model in different districts/geographic areas to generate learning and facilitate national scale-up in the three countries.
3. In phase 2, to support the national governments in the three countries to further scale up iKMC at sub-national and national levels.

The study will also obtain costs of setting up M-SNCUs in each context to support countries in decision making for scale up.

### 2.2 Study design

**Figure 1.**
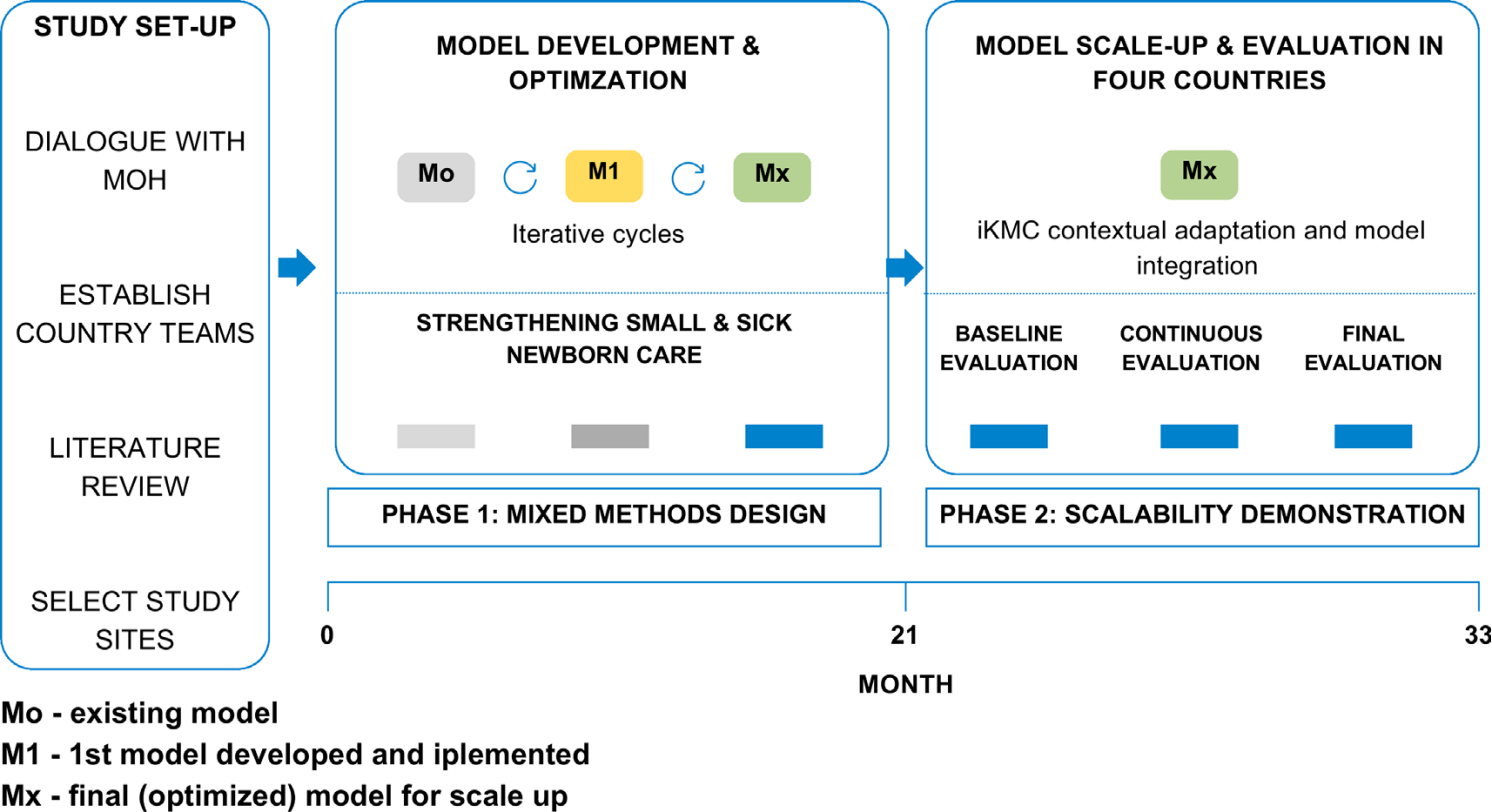
Overview of the study.

This is a multicountry implementation research and effectiveness study that will be conducted in the context of improved care for preterm and/or LBW infants. The first phase of the study will begin with a model development and optimisation phase, using a mixed-methods design that applies qualitative and quantitative research methodologies. Formative research will be used to identify barriers and facilitators for quality iKMC in designing the initial implementation model. Concurrent programme learning using qualitative research and outcome measurement while implementing the model in routine care settings will be used to improve the model iteratively until high coverage of quality iKMC is achieved. Figure 1 below illustrates the overview of the phase 1 study design. In the second phase of the study, the scalability of the final (optimized) implementation model will be demonstrated in additional health administrative areas in each country.

### 2.3 Setting of the study

***2.3.1 Study countries***

In phase 1, the study will be conducted in six sites, two in Africa (Ethiopia and Nigeria) and four in Southern Asia (Bangladesh and India), and in phase 2 the study will be conducted in three countries (Ethiopia, India and Nigeria). These regions have a high burden of LBW and neonatal mortality; they contribute substantially to the global burden. Southern Asia alone accounts for nearly half of all LBW infants born globally and Africa for approximately 25%. ^12^^,^^13^ There is strong commitment from the governments in these countries to improving hospital-based care for preterm and/or LBW infants.

***2.3.2 Selection of study sites within the country***

Each site will base its study in one or more health administrative areas (called ‘district’ in Bangladesh and India; ‘woreda’ in Ethiopia and ‘local government area’ in Nigeria). All sites will implement the study in areas that have: (i) population size of about 1-2 million for South Asian countries and half a million to a million for African countries; (ii) neonatal mortality and LBW rates which are the same or higher than national levels; (iii) institutional birth rates that are the same or higher than national levels (iv) quality of care consistent with local or national hospital standards; (v) at least one hospital/facility with a level 2 newborn care unit or a facility where it is feasible to set up one; and (vi) strong commitment of the facility and local government to sustain iKMC after the study ends. The study districts will be selected jointly with the leadership of the ministries of health and their local subsidiaries. Table 1 details the study site names, principal investigators and iKMC implementing facilities in phase 1. Phase 2 site selection will be undertaken through consultations with multiple stakeholders across the three participating countries.

**Table 1.** Description of study sites, Principal Investigators and iKMC implementing facilities for Phase 1.

Country and Site	Name of Principal Investigators, affiliations	IKMC implementing facilities
** Bangladesh** – Rajshahi District, Rajshahi Division	Ahmed Ehsanur Rahman, International Centre for Diarrhoeal Disease Research, Bangladesh	Rajshahi Medical College Hospital
** Bangladesh** –Manikganj District, Dhaka Division	Salahuddin Ahmed, Projahnmo Research Foundation, Bangladesh	1. Bedded General Hospital 2. Manikganj Medical College Hospital 3. Mono Medical College
**Nigeria** – Osogbo, Osun state	Ebunoluwa Adejuyigbe Department of Paediatrics, Obafemi Awolowo University, Ile-Ife, Nigeria	Osogbo State Hospital
**Ethiopia** – Sidam Region, Hawassa	Abiy Seifu Estifanos, Department of Reproductive, Family and Population Health School of Public Health, Addis Ababa University, Ethiopia	1. Hawassa University Comprehensive Specialized Hospital 2. Adare General Hospital 3. Tula General Hospital 4. Busholo MCH specialty centres
** India** – Ambala, Haryana state	Sarmila Mazumder, Society for Applied Studies, India	Ambala District hospital
** India** – Ghaziabad, Uttar Pradesh state	Aarti Kumar, Community Empowerment Lab, India	Ghaziabad District hospital

***2.3.3 Characteristics of participants***

**Inclusion criteria**

**Figure 2.**
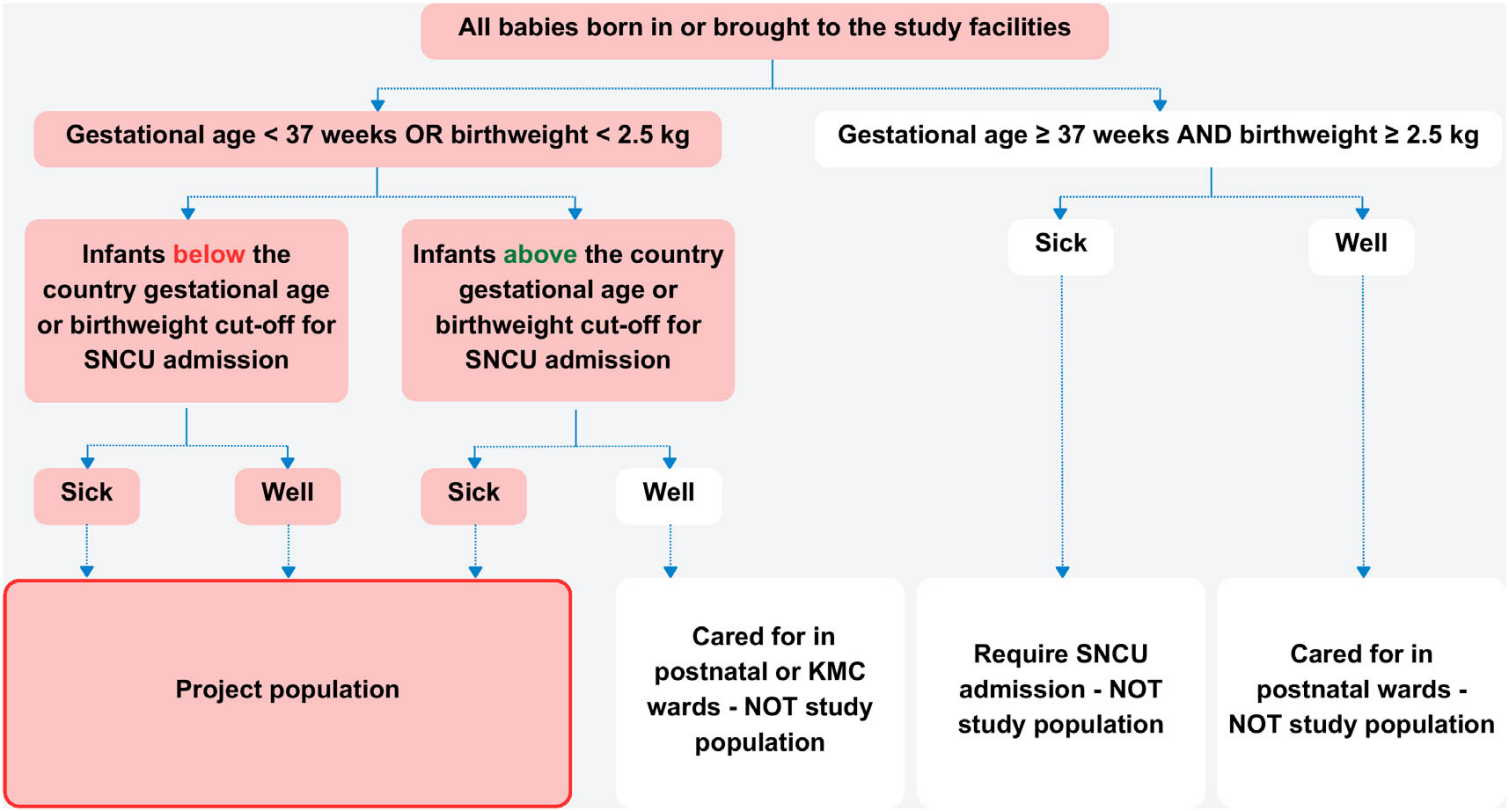
Description of study population in the iKMC implementing facility.

The study population is preterm and/or LBW infants (gestational age <37 weeks or birthweight <2.5 kg) requiring care in the level-2 newborn care unit and their mothers, i.e., infants who are below the country-specific cut-off point for birthweight or gestational age for level-2 newborn care unit admission, or those preterm and/or LBW infants who are above the country-specific cut-off but are sick and need admission ( Figure 2). However, the strengthening of care will be done for all small and/or sick newborns irrespective of where they are cared for in the health facility.

Exclusion criteria*

Preterm and/or LBW infants requiring care in the level 2 newborn care unit who are critically sick, for example:

• are not breathing spontaneously (after resuscitation) at 60 minutes after birth
• have congenital malformations that interfere with the intervention, or the intervention interferes with the required care for the congenital malformation (e.g., anencephaly, congenital heart disease, gastroschisis, hydrocephaly, multiple malformations, omphalocele, tracheoesophageal fistula, etc.) or
• are in shock (in need of inotropes)
• received invasive mechanical ventilation in the first 2 hours of birth for inborn babies or within two hours of admission to level 2 newborn unit for outborn babies or
• liveborn who died in the first 2 hours of birth or first 2 hours of admission or were dead at the time of admission to the iKMC implementing facility

*These infants will be excluded from the study for outcome measurement purposes however all newborns will receive appropriate care in the iKMC implementing facility.

WHO 2022 guidelines include a recommendation for KMC for all preterm and/or LBW infants starting immediately after birth unless the infant is critically sick. Therefore, KMC provision for all preterm and/or LBW infants will be strengthened at all levels of care. However, the purpose of the study is to implement and scale up iKMC for preterm and/or LBW infants who are at high risk of being separated from the mother, i.e., those preterm and/or LBW infants who require SNCU care, either because they are too small or sick. Hence, while the facilities provide iKMC for all preterm and/or LBW infants based on the WHO recommendation, the study will include only preterm and/or LBW who require SNCU admission after birth.

***2.3.4 Description of study team***

The national and provincial ministries of health are the most important partners in this implementation research effort. WHO ensures that the national, provincial and district level programme managers are engaged in the design and implementation of the study. Another important stakeholder is the local research group, which has the skills and experience to conduct the study. This research group has been jointly identified by the ministry of health and the WHO national office in each country.

The existing health workforce who are already part of the health system in the study site, programme managers and researchers jointly make up the study team. The iKMC intervention is delivered by the health workforce; the main role of the research team is to support the implementation of the intervention, and data collection for iterative programme improvements and programme evaluation.

The research team consists of three small independent teams: (i) **programme learning team**, trained in interviewing techniques and qualitative methods will monitor activities for iKMC implementing facilities and hold discussions with key stakeholders to capture their perceptions of what is and what is not working. This team is involved with formative research and programme learning and is helping to formulate and improve the intervention; ii) **implementation support team** guides the health workforce and support the creation of an enabling environment to implement the intervention in iKMC implementing facilities through process monitoring; and (iii) **outcome measurement team,** will independently measure the outcomes in the study population. Table 2 describes the study teams.

**Table 2.** Description of study teams.

	Government partners	Research team		
Overall role	Decision-making and implementation	Implementation, qualitative and quantitative research data collection and evaluation of the implemented programme		
Staff	Policy makers and health staff/health care providers	Formative research and programme learning team	Implementation team	Outcome evaluation team
Activities	Decide initial and subsequent implementation models Implement activities in the implementation model. Responsible for all resources necessary. Management and administrative support for implementation, training on standard protocol, Monitoring from national level. Facilitate institutionalization for accountability, sustainability, and scalability.	Formative research to develop the initial model. Continued programme learning data to help refine and optimize implementation model. Monitor process outcomes. Contribute to discussions to refine and optimize model.	Support policy makers, programme managers, and health staff/health care to implement the model. Ensure complete, valid documentation of service in the register. Facilitate preparedness such as infrastructure, training, logistic arrangement including job aid, communication materials. Contribute to refinement and optimisation of the model.	Collect quantitative data on outcomes including coverage of iKMC before, during, and after the development of optimized implementation model. Contribute to discussions to refine and optimize model.

### 2.4 The iKMC implementation model

***2.4.1 Key components of the iKMC implementation model***

• The key components of the iKMC implementation model includes the following interventions. Skin-to-skin contact is initiated within 2 hours after birth if the infant is born within the iKMC implementing facility; if the infant is born outside, then within 2 hours of reaching the iKMC implementing facility (for those infants who reach the facility within 24 hours of birth)
• Continuous skin-to-skin contact is provided by a mother/surrogate for at least 8 hours per day during the stay in the level 2 M-SNCU (average hours per day for the overall M-SNCU stay)
• Support for early and exclusive breastmilk feeding is provided to the mother
• Required medical care for the mother and infants is provided without separation, as much as possible

***The pre-requisite for the intervention***

**Figure 3.**
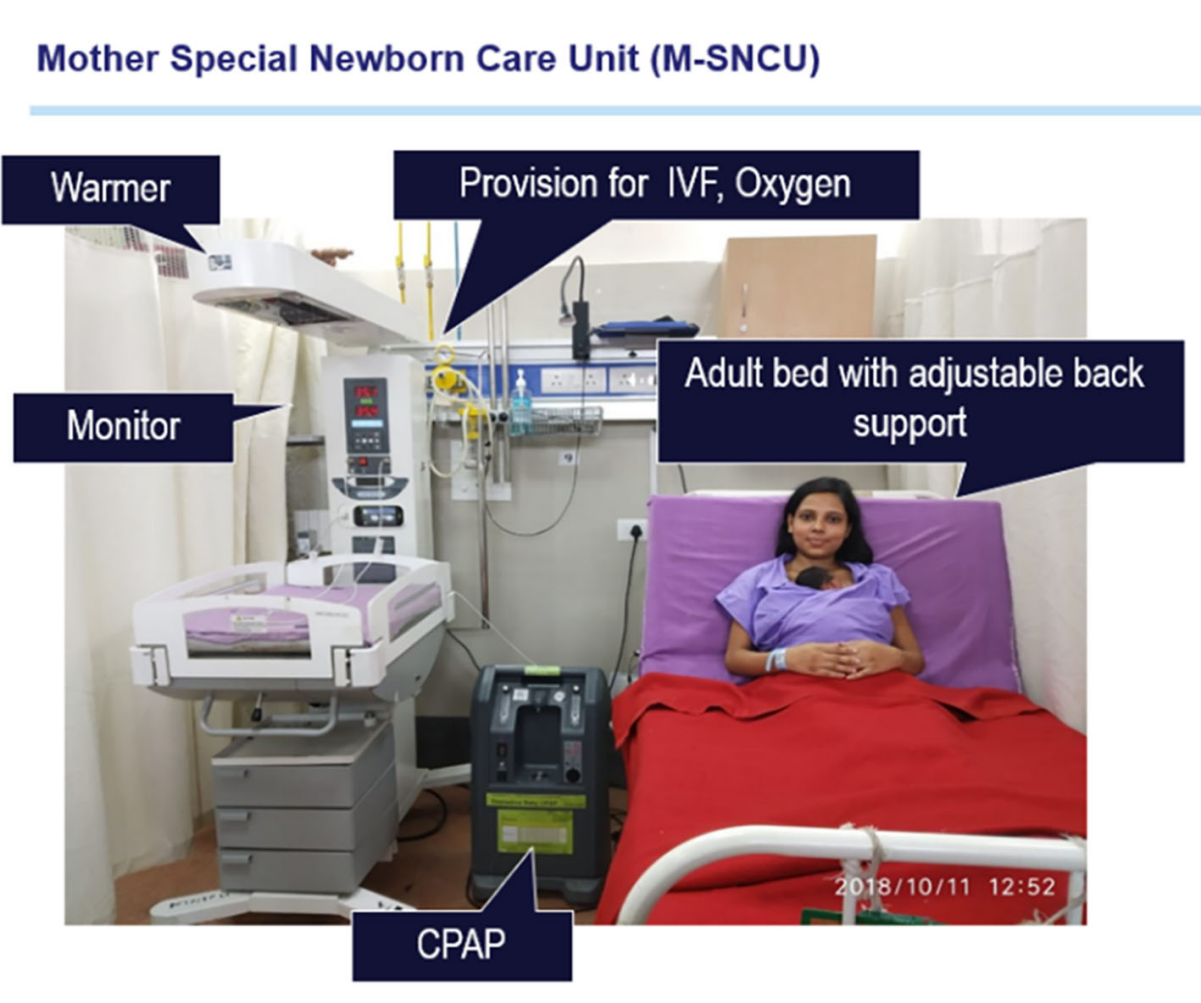
M-SNCU with level II newborn care facilities. Figure reproduced with permission from Ref. 18.

**Figure 4.**
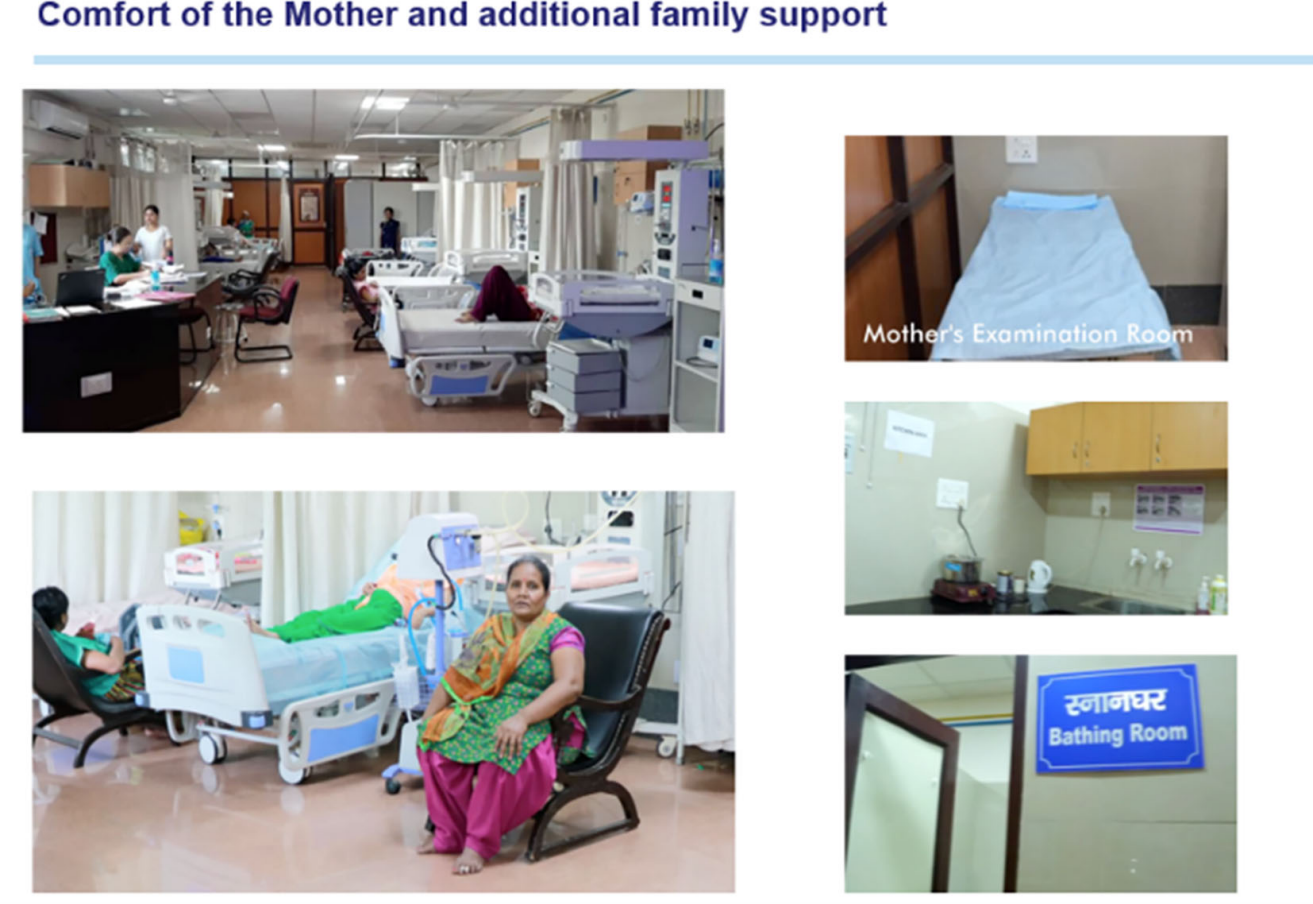
Enabling environment for mothers (Examination room, pantry and bathing facilities). Figure reproduced with permission from Ref. 18.

➢ ***Converting the level 2 SNCU into a level 2 Mother-SNCU (M-SNCU),*** is a key requirement to enable the provision of iKMC. This means transformation of the SNCU to a Mother-SNCU (M-SNCU) to allow the mother or surrogate to stay with the infant 24/7. The nomenclature of SNCU may vary across sites—SCANU in Bangladesh, SCBU in Nigeria, NICU Ethiopia, and SNCU in India—but for consistency, SNCU, referring to level 2 newborn care unit, will be used throughout this document. The organization of services described below builds on the learnings from the iKMC RCT study ( Figure 3).
➢ A typical level 2 M-SNCU will have a reclining adult bed and a reclining chair for the mother/surrogate, a warmer for the infant, provision for intravenous fluids, oxygen, CPAP support and pulse oximeter for continuous monitoring. In addition to iKMC, a minimum package of care for preterm and/or LBW infants requiring care in the SNCU will be provided ( Table 3). Core components of the intervention package will include support to empower and enable the mother to take care of her infant and to ensure the appropriate medical care of the mother and infant, while keeping skin-to-skin contact as long as possible. Health workers will be trained to provide clinical care to mothers and infants, as well as counselling and support to mothers and caregivers. Mothers are always free to determine if they wish to provide skin-to-skin contact and breastfeeding, and if they would like to designate a surrogate.
➢ All infrastructure, equipment, and staff required to provide high quality comprehensive care for the sick and small newborns, including respiratory support with CPAP, optimal nutrition with emphasis on mother’s own milk feeding, adequate monitoring and protection of infants will be strengthened by government partners with technical support by the research team. Concurrently, postnatal and KMC wards in the iKMC implementing facility will be strengthened to enable the provision of KMC for preterm and/or LBW infants who do not require admission into the M-SNCU ( Figure 4).
➢ Initiation of skin-to-skin contact as soon as possible after birth, promotion and support for prolonged skin-to-skin contact up to 24 hours per day.
  As mentioned above, mothers will be counselled and supported to initiate continuous skin-to-skin contact immediately after birth, or on presentation at the iKMC implementing facility if born outside - provided they reach the facility within 24 hours of birth - aiming for at least 8 hours of skin-to-skin contact per day. Initiation of KMC does not have to wait for the infant to be clinically stable. SSC care will be initiated by the mother or the surrogate within the birthing room or the operation theatre, continued during transfer and stay in the M-SNCU. The mother/surrogate and infant will be kept in the M-SNCU until the infant is stable enough to be transferred to the KMC ward/postnatal ward or any other ward in the iKMC implementing facilities (for stable infants), and KMC will continue to be provided by the mother until they are discharged from the hospital. At the time of discharge, the mother will be advised to continue KMC at home.
➢ ***Promotion and support for early and exclusive breastfeeding/breastmilk feeding***
  Mothers will be counselled and supported to put the infant to the breast and express milk when they are in the M-SNCU. Even if the infant is unable to feed from the breast, putting the infant to the breast provides the infant the opportunity to learn how to attach and suckle and stimulate milk production. Continuous skin-to-skin contact between mother and infant is likely to facilitate breastfeeding. Mothers and/or surrogates will receive counselling to promote and support exclusive breastfeeding. If the infant is unable to feed from the breast, breast milk feeding using a cup, or a tube will be supported. This breastfeeding support will continue through the length of stay in the hospital, including in the KMC ward. At the time of discharge, the mother will be advised to continue exclusive breastfeeding at home. If a mother is deceased or unable to express breastmilk due to medical reasons, the team will explore alternative feeding for the infant based on the national guideline (e.g., donor milk or formula feed).
➢ ***Essential/routine postnatal care for the mother and specialized care for the infant, provided without separation and with respect***
  The mother and infant will be provided health care without separation as much as possible. Mothers will be provided health care by midwives, nurses, and obstetricians while she is in the M-SNCU. If a mother has any complication for which she needs to stay in or be transferred to the adult intensive care unit or operation theatre, skin-to-skin contact will be initiated and/or continued with a surrogate/caregiver. If the infant requires a procedure or treatment that is not possible during skin-to-skin contact, the infant will be shifted to an incubator or radiant warmer. Then, skin-to-skin contact will be temporarily interrupted for the period of the procedure or treatment and resumed as soon as possible thereafter. Health workers will have received orientation and will be supported to provide respectful care to mothers and/or surrogates and their infants.
➢ ***Strengthening the linkages within the hospital across departments***
  Any preterm and/or LBW infant identified at any point of care, including the emergency ward, paediatrics outpatient department (OPD), labour room and operating theatre, or accompanying the mother in the gynaecology/obstetric OPD for any postnatal problem, will be assessed for special care requirements and directed to the M-SNCU or KMC ward accordingly. Staff at each of these contact points will be oriented and trained to identify infants who require admission to the M-SNCU. To avoid delays due to admission processes, staff will be trained so that they can initiate KMC wherever feasible.
➢ ***Strengthening the referral pathway and appropriate identification of newborns who require M-SNCU care***
  To achieve population level coverage of iKMC, linkages with birthing facilities without a SNCU are crucial. Care will be strengthened, ensuring that infants are accurately weighed within one hour of birth, and infants who meet the M-SNCU admission criteria will be referred immediately to a hospital with an M-SNCU, based on national guidelines for referral. The staff will be trained and motivated to initiate KMC and ensure that infants are transported in skin-to-skin contact with the mother or surrogate. Strengthening the referral pathway will include facilitated decision making by the family, facilitated transport, addressing financial barriers through local government support, and increased readiness and preparedness of the M-SNCUs to receive the referrals. Sub-district and lower-level facilities will also be strengthened to provide KMC to preterm and/or LBW infants who are stable and do not meet the referral criteria based on each country’s guideline. After discharge from the hospital, the mother and family will be introduced to a community health worker (CHW), or the relevant health cadre in each country, who can provide counselling and support for continued KMC at home.
  Strengthening timely and appropriate identification and referral is important for high coverage, particularly for infants born at home.
  Various potential strategies will be explored, including but not limited to: social and behavioural change communication activities to create awareness and generate demand; more frequent follow up of pregnant women who are at risk of giving birth to preterm and/or LBW infants as identified during antenatal visits; motivation of families and communities to inform CHWs when women go into labour or as soon as possible after birth; training of CHWs in appropriately weighing and identifying LBW infants, and strengthening transport and referral for those preterm and/or LBW infants born at home. Strategies will be discussed and refined through co-design workshops, and final decisions will be taken by relevant stakeholders based on the implementation learning and outcomes.
  Strengthening care for small and/or sick newborns:
  High-quality small and/or sick newborn care reduces neonatal mortality, prevents complications, and supports optimal growth and development. It requires a strong health system, trained providers, and well-integrated community and facility-based services. WHO’s standards for improving the quality of care for small and/or sick newborns ^14^ emphasize:
• Clinical management of conditions such as respiratory distress, infections, hypothermia, and feeding difficulties.
• Well-equipped facilities with CPAP machines, incubators, radiant warmers, and essential medications.
• Competent, multidisciplinary healthcare teams, including trained nurses, midwives, and paediatricians
• Family-centered care, promoting KMC, parental involvement, and respectful communication.
• Functional referral systems ensuring seamless transitions across different levels of newborn care

**Table 3.** Every newborn action plan (ENAP) health system level for newborn care organizing services by level of care (Inpatient care for small and sick newborns: requirements for care at different health system levels).

Level	Type of care provided	Health system requirements		Standards of care & evidence-based interventions
Primary	Essential newborn care	Place	• Space for childbirth, with specific areas for postnatal care for mother and baby to stay together • Infrastructure for handwashing • Outpatient facility for routine postnatal care and management of newborn problems	• Immediate newborn care (through drying, skin-to-skin contact of the newborn with the mother, delayed cord clamping, hygienic cord care) • Neonatal resuscitation for those who need it • Early initiation and support for exclusive breastfeeding • Routine care • Vitamin K, eye care and vaccinations, **weighing** and clinical examinations • Prevention of mother to child transmission of HIV • Assessment, management and referral of: - Bacterial infections including possible severe bacterial infection (PSBI), where referral is not possible - Jaundice and diarrhoea - Feeding problems - Birth defects and **other problems** • Pre-discharge advice on mother and baby care follow up • Referral of women who are high risk preterm or LBW baby to a higher-level hospital for childbirth • Skin-to-skin contact with the mother or surrogate
		People	• Skilled attendance 24/7 (e.g. midwifery and nursing staff +/- doctors) • Support staff for cleaning	
		Health technologies	• Linen/towels for drying and wrapping • Bag and mask resuscitation • Radiant heater, warmth source • Thermometer • Equipment for clean cord care • Vitamin K, eye ointment, • Weighing digital scale, tape • Immunization commodities • Antibiotics • Oxygen • Pulse oximeter	
		Support system	• Water, sanitation & hygiene (WASH) and infection, prevention and control • Communication and functional referral system • Newborn patient record and facility register • Written policy for zero separation • Easy access to fathers/caregivers	
Secondary	Special newborn care	Place	• A dedicated warm space of a facility with specific areas for resuscitation, stabilization and care • Dedicated area for KMC • Accommodation for mothers (space for mothers to room in and stay with their baby) • Electricity supply (e.g. generator back-up) • Infrastructure storage for human milk	• Thermal care • Comfort and pain management • Kangaroo mother care including follow-up * • Assisted feeding for optimal nutrition (cup feeding and nasogastric feeding) • Safe administration of oxygen • Prevention of apnoea • Detection and management of neonatal infection • Detection and management of hypoglycaemia • Detection and management of jaundice • Detection and management of anaemia including blood transfusion • Detection and management of neonatal encephalopathy • Seizure management • Safe administration of intravenous fluids • Detection and referral management of birth defects • Immediate kangaroo mother care • Transition to intensive care • Continuous positive airway pressure ** • Exchange transfusion * • Detection and management of necrotizing enterocolitis (NEC) ** • Specialized follow up of high-risk infants (including preterm)
		People	• Specialized nursing and midwifery staff 24/7 • Doctor with neonatal skills on call • Support staff (nursing auxiliary and cleaning staff )	
		Health technologies	• Syringe pump and accessories (e.g. neonatal cannula) • Feeding equipment (nasogastric tubes and cups/spoons) • Basic diagnostics (e.g. glucometer, urine dipsticks) and micro-methods • Medicines (e.g. antibiotics, caffeine, IV fluids, phenobarbital) • Mobile X-ray system • Warmers and cots • Effective phototherapy equipment (e.g. LED) • Continuous positive airway pressure	
		Support system	• 24/7 access to the facility for mothers and caregivers • Facilities for bathing, laundry and cooking/food • Clinical charts and facility registers	
Tertiary	**Intensive newborn care**	**Place**	• **Designated intensive care ward** • **24/7 uninterrupted electricity** • **Space for mothers to room in and stay close to their baby**	• Advanced feeding support (e.g. parenteral nutrition) • Mechanical/assisted ventilation including intubation • Screening and treatment of retinopathy of prematurity • Surfactant treatment • Investigation and management of birth defects • Paediatric surgery • Genetic services
		People	• Nurses with specialized competencies in neonatal care 24/7 • Doctors with specialized competencies in neonatal care 24/7 • Neonatologist on call • Other doctors with specialized competencies in neonatal care • (anaesthetics, surgery, radiology, cardiology, neurology, ophthalmology) • Allied health professional (physiotherapy, nutrition, speech therapy, occupational therapy, audiology, etc.)	
		Health technologies	*In addition to special care equipment and commodities* • Intermittent positive-pressure ventilation, high flow oxygen via nasal cannula • Monitoring equipment • Surfactant therapy • Advanced medicines • Supplies for advanced nutrition support (e.g. total parenteral nutrition) • Specialist equipment and accessories	
		Support system	• 24/7 advanced laboratory support and other diagnostics including medical imaging • Transport and safe referral if needed • Hospital information management system	

We will focus on the interventions, within the study’s scope, highlighted in **underlined bold text** .

* Outpatient care.

** The interventions listed under special care mark a transition to intensive care. Hospitals providing special care should introduce these interventions before upgrading to intensive care.

***Frameworks to guide the development and optimisation of an iKMC implementation model***

We will use *Consolidated Framework for Implementation Research* ^15^ which recognizes the non-linear and recursive nature of the implementation process. We strongly believe that adapting implementation based on process learning and the concurrent evaluation of coverage and quality is critical to achieving the optimal models. Throughout the implementation process, we will consider factors, strategies, and outcomes that will influence the development and optimisation of an iKMC implementation model. The Consolidated Framework for Implementation Research is composed of the following five domains, with multiple constructs related to each domain:

1. ***Characteristics of individuals involved:*** This domain refers to characteristics of the individuals who would be key for implementation of the intervention, which include preterm and/or LBW ***infant*** s, mothers, parents and families, surrogates, CHWs, midwives, nurses and doctors, facility managers, and programme managers at district level, provincial, and national levels. The relevant constructs related to this domain include knowledge and beliefs about the intervention, self-efficacy, and individuals’ motivation for change. These will all be explored during formative research and appropriate elements will be added to the implementation model.
2. ***Intervention definition and characteristics:*** This domain refers to characteristics of the intervention, which in this case will be iKMC. The specific barriers and facilitators to the implementation of the core elements of the intervention will be identified during formative research and the implementation model will be designed and adapted accordingly.
3. ***Outer setting or broader context:*** This domain refers to the economic, political, social, and cultural context which would influence the implementation of iKMC. The relevant constructs related to this domain include resources for the care of preterm and/or LBW infants, relevant policies, political support, barriers, and enablers for efforts for newborn survival and KMC, societal norms and perceptions about care of the small infants and KMC. During the formative research, we will review national, subnational and facility policies and standards to understand what policy changes need to be introduced to ensure implementation of iKMC. The cultural context that influences iKMC will also be explored.
4. ***Inner setting or health system context:*** This domain refers to the health system, and health facility, and the context which would influence implementation of iKMC, including setting up M-SNCUs. The relevant constructs related to this domain include structural characteristics of the facilities, human resources and organization of care for preterm and/or LBW infants, culture (overarching thinking about care of the small infants), implementation climate (need for change, compatibility with the intervention, relative priority, motivations, goals and feedback and learning climate), and readiness for implementation (leadership engagement, available resources, and access to information, knowledge and skills for implementing iKMC).
5. ***Process of implementation:*** This domain of the framework focuses on engaging the relevant stakeholders, executing, reflecting, and evaluating with the aim of optimizing the implementation model for iKMC. The process is described in detail in the sections below.

## 3. Model optimisation and development (Phase 1)

In the first phase of the study, the model optimisation and development process will begin with formative research to gather insights on the needs and challenges faced by health providers and families in the four countries. Based on the findings of this research, study teams and stakeholders will collaboratively develop an initial iKMC model that addresses the specific barriers to implementation. This initial model will then be refined through iterative implementation cycles, data collection, and documentation of programme learning, to develop a model that will be scaled up during the second phase of the study.

### 3.1 Formative research

The formative research will focus on identifying and understanding barriers and enablers to implementing iKMC in the local context. This will include qualitative and quantitative aspects, with the overall aim to inform a user-centric design of the intervention model. The qualitative component will examine the needs, motivations, and influencers of the mother-infant dyad, while the quantitative aspect will assess the readiness of facilities, including infrastructure and services, and will perform a preliminary assessment of SSNC at secondary-level facilities in the study area. The formative study will occur in a sample of health facilities and community catchment areas across the six sites where iKMC interventions will be developed.

The specific objectives of the formative research phase are:

1. To understand perceptions of women regarding the care for preterm and/or LBW infants, including their care-seeking practices, the barriers and facilitators to accessing health facilities, the quality of care received, their experiences with skin-to-skin care and breastfeeding, their concerns about caring for their infants, the support they need, and the acceptability of the intervention;
2. To explore family and community norms, perceptions, and values regarding the care of preterm and/or LBW infants, how to build family and community support for facility births, referrals to iKMC facilities, and the care of preterm and/or LBW infants in general, as well as for the implementation of iKMC;
3. To explore health workers’ knowledge, norms, and skills around care for preterm and/or LBW infants, as well as their perceptions about the feasibility and acceptability of different components of the iKMC implementation model;
4. To identify and understand factors affecting the implementation and scale-up of iKMC, including existing policies and standards, current practices in the care for preterm and/or LBW infants, and the feasibility and acceptability of different components of the iKMC implementation model;
5. Identify and agree with stakeholders on strategies that address barriers and enablers to implement the iKMC model effectively.
6. Map SNCU facilities to assess their readiness for implementing the WHO minimum package of care for small and sick newborns.

The following activities will be conducted in the formative phase:

i. Qualitative in-depth interviews (IDIs) with policy level managers/administrators at local and national level, family members, women who had preterm birth and/or LBW infants who were cared for in a study facility in the last month;
ii. Focus group discussions (FGDs) with women in the community and female family members, including those who gave birth in the last 12 months in a study facility or at home;
iii. FGDs with men in the community, including partners of women who gave birth in the last 12 months in a study facility or at home;
iv. Small group (2-3 people) or individual interviews with traditional birth attendants, women and community group leaders, and other community stakeholders;
v. Small group (2-3 people) or individual interviews of health workers (CHWs, midwives, nurses, doctors, obstetricians, neonatologists, paediatricians, and facility management);

The qualitative study will take place in a sample of health facilities and community catchment areas across the six sites where iKMC interventions will be developed. To enhance rigor, data collection will be conducted by trained researchers using standardized tools, with concurrent analysis and triangulation across participant groups and data sources to support credibility of findings.

### 3.2 Facility mapping

Existing SNCUs will be evaluated to assess the facility’s readiness to provide a minimum level of care to preterm and/or LBW infants, including KMC. It will include an assessment of the availability of:

i. Adequate infrastructure (e.g., closed thermoneutral space with handwashing facility, private space for KMC),
ii. Human resources (number and type of staff, e.g., doctors and nurses trained in newborn care),
iii. Equipment and supplies (e.g., CPAP for respiratory support, pulse oximeters to ensure safe oxygen use),
iv. Standard operating procedures and protocols for newborn care (e.g., infection prevention and control including antibiotic administration, fluid management),

The findings from these activities will inform the development of an initial iKMC implementation model in participating sites. Through co-design workshops, stakeholders will collaborate to define the activities and interventions that will constitute the model.

### 3.3 Preparatory activities at different levels

The study team will conduct preparatory activities in selected districts (administrative area), particularly in the selected hospitals where iKMC is to be implemented while formative research will be conducted. An important part of these preparatory activities is to strengthen implementation of a minimum package of care for preterm and/or LBW infants requiring care in SNCUs (see Table 3).

The WHO iKMC trial identified two key common gaps in care: the provision of appropriate respiratory support and periodic clinical monitoring of infants. These gaps will be covered by efforts to ensure an adequate number of safe CPAP machines and pulse oximeters are made available at all iKMC implementing facilities. In addition, standard operating procedures for the use of this equipment will be developed and introduced. To ensure high quality and coverage of KMC for stable infants, learnings from the scale up KMC study will be implemented. ^16^

The following activities are envisaged prior to the implementation of iKMC in M-SNCUs:

1. Activities aimed at maximizing access of preterm and/or LBW infants to iKMC-implementing facilities including accurate birth weight recording and referral of preterm and/or LBW infants born at home or lower-level facilities that do not provide KMC including skin-to-skin contact.
2. Activities aimed at initiating iKMC and maintaining KMC for stable preterm and/or LBW infants who were born in or referred to the facility in SNCU through changes in infrastructure and training, motivation and support of facility staff. The SNCU for those require admission, will be strengthened to provide effective interventions in the standard of care, including optimal use of CPAP, breastmilk feeding, prevention and treatment of infections, and monitoring.
3. Activities aimed at promoting birth at a health facility. Activities will be undertaken to strengthen referral and transport. Health education activities will be strengthened to promote births in facilities and to increase awareness of the needs of preterm and/or LBW infants. In an area where home birth is high, CHWs will promote immediate care seeking at iKMC implementing facilities in case of preterm and/or LBW infant is born. Community level activities may also support the continuation of KMC at home after discharge even if this is beyond the scope of the study; these would include telephone contact by hospital staff with CHWs and families and home visits by trained CHWs. This network of primary and secondary level facilities and community will transform maternal and newborn care into the study implementation area, including the care of the small and sick newborns.

### 3.4 Development of the iKMC implementation model

***3.4.1 Initial model***

**Figure 5.**
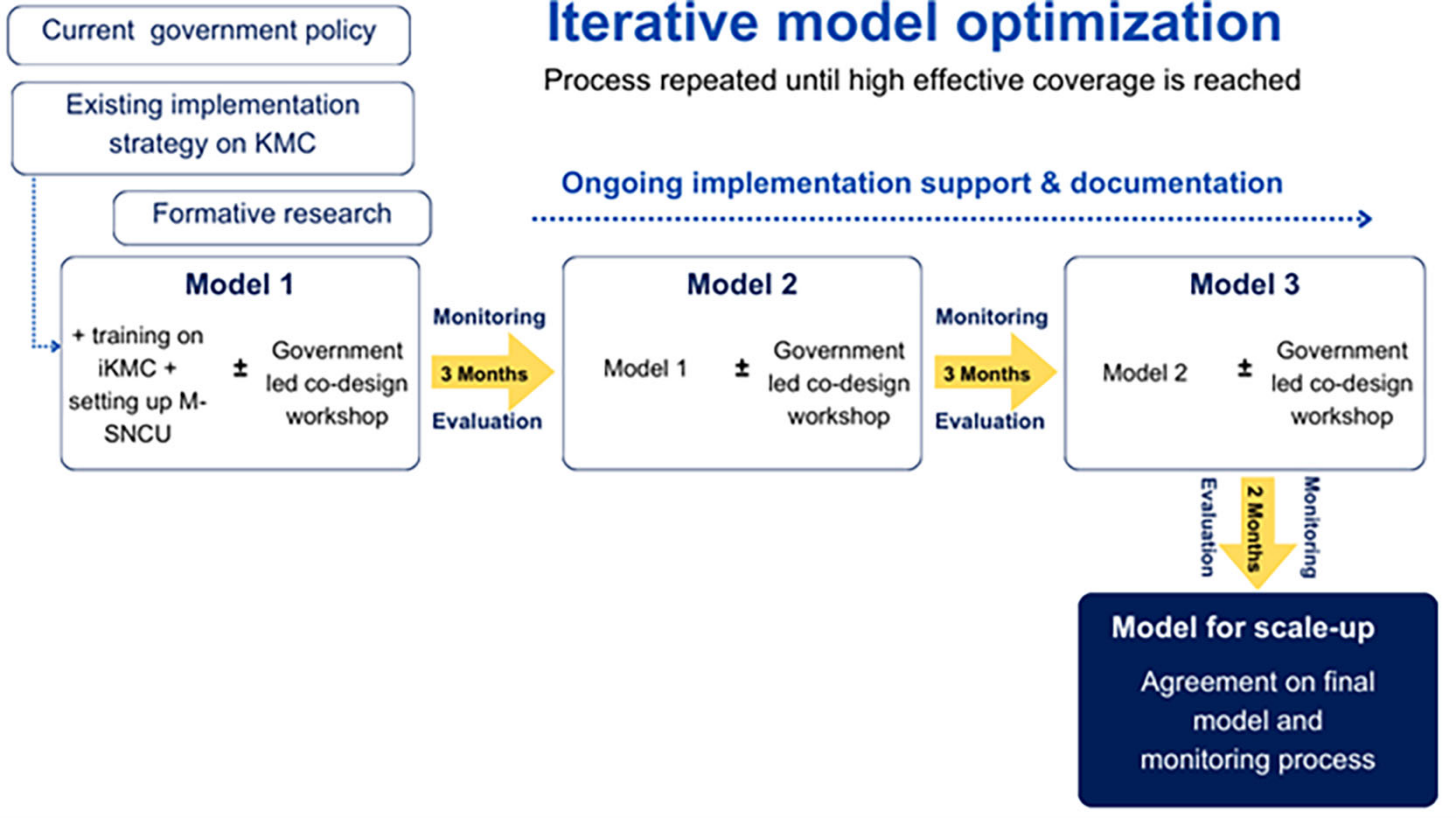
Iterative process of model development.

Based on the findings of the formative research, an initial model (Model 1 in Figure 5) of the implementation of iKMC will be developed for implementation within the existing health system. This will be done in co-design workshops which will be held in each country under the leadership of national/provincial ministry of health, and attended by the research group, district and hospital managers, doctor, nurse, and midwife leaders in participating hospitals, CHWs and women’s and parents’ groups representatives, and the WHO technical support team. Group discussions will be held to inform the implementation strategy using the APEASE criteria (Affordability, Practicability, Effectiveness, Acceptability, Safety, and Equity). ^17^ A similar methodology will be used during subsequent re-design workshops (see below).

***3.4.2 Model iterations***

Implementation of Model 1 will be initiated with concurrent programme learning/monitoring as well as quantitative data collection on coverage and quality outcomes. The quantitative coverage and quality data are the parameters for optimisation of the implementation model, while the qualitative programme learning data (through interactions with managers, healthcare workers, CHWs and women and families to understand their perspective on implementation) provides answers to why we are achieving or not achieving the desired outcomes.

As mentioned above, the implementors are routine health system staff, supported by a small implementation support team. The two other teams, a programme learning team and an outcome measurement team, are respectively collecting the above-mentioned qualitative and quantitative data. The programme learning team frequently shares data with the implementors and implementation support teams to continuously improve implementation.

To ensure the effective implementation of the initial iKMC model and to observe changes, such as in iKMC coverage, it is important to plan and document how core strategies will be executed over a three-month period. For example, the first two to three weeks of the initial month could be dedicated to implementing core activities like health provider training, mentorship, ensuring equipment and consumables such as CPAP and KMC binders, and improving referral systems with facilities in the network of care. The remaining time can then be used for follow-up support and supervision, including ongoing mentorship and programme monitoring.

After the implementation of the initial model for 3 months, a re-design workshop led by the Ministry of Health will be organized. At this meeting, lessons from implementation and data from programme learning and coverage and quality measurement will be used to review and refine the implementation model. The re-design workshop would aim to refine, improve and revise the implementation model to move towards higher coverage and quality of the iKMC implementation model. A period of 3 months provides adequate volume and range of implementation experience with qualitative and quantitative data to support decisions on changes of the implementation model. However, this period will vary among the sites based on contextual needs.

***3.4.3 Model optimisation***

**Figure 6.**
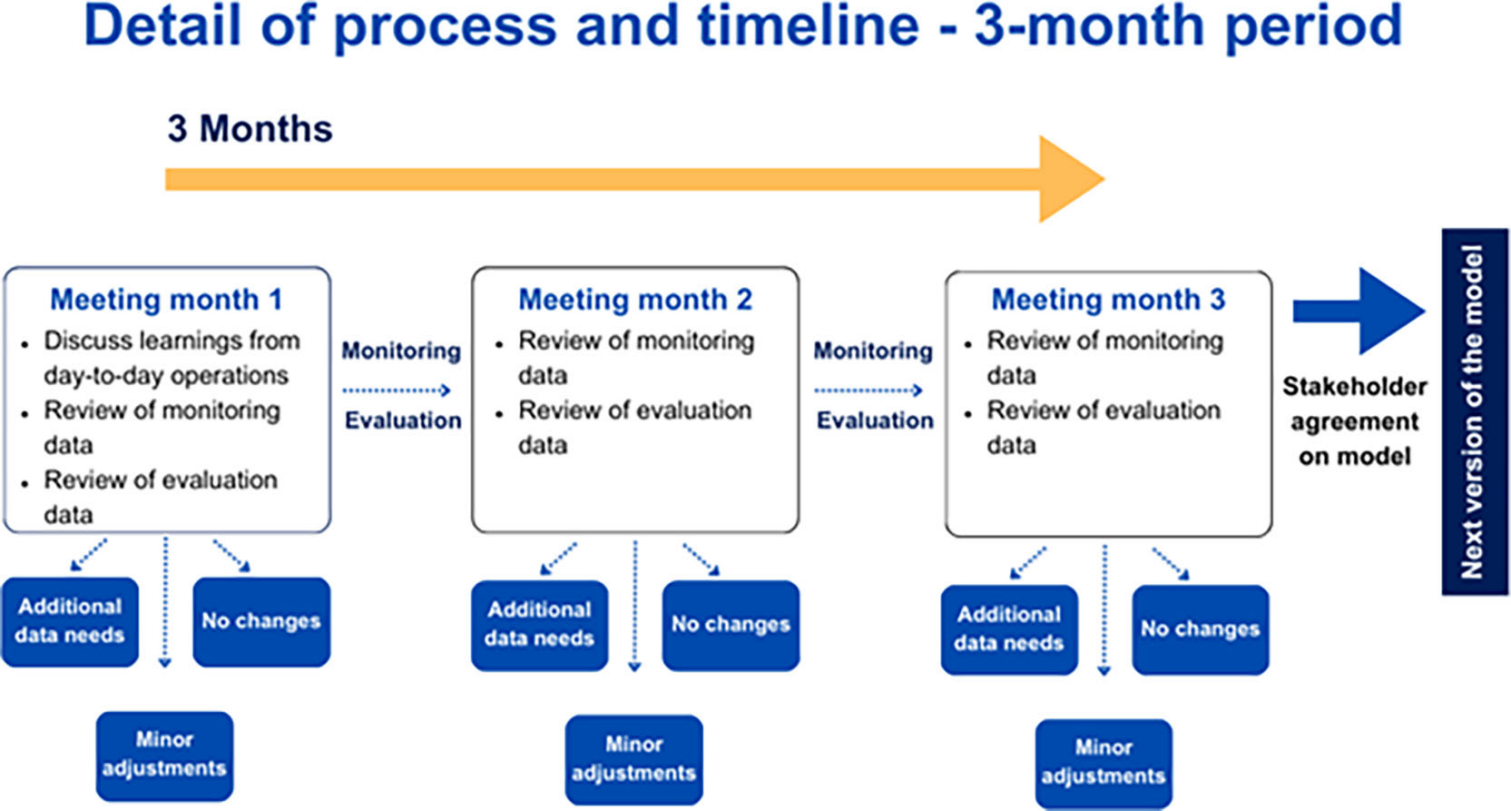
Model optimisation: process with timeline – 3 month period.

**Figure 7.**
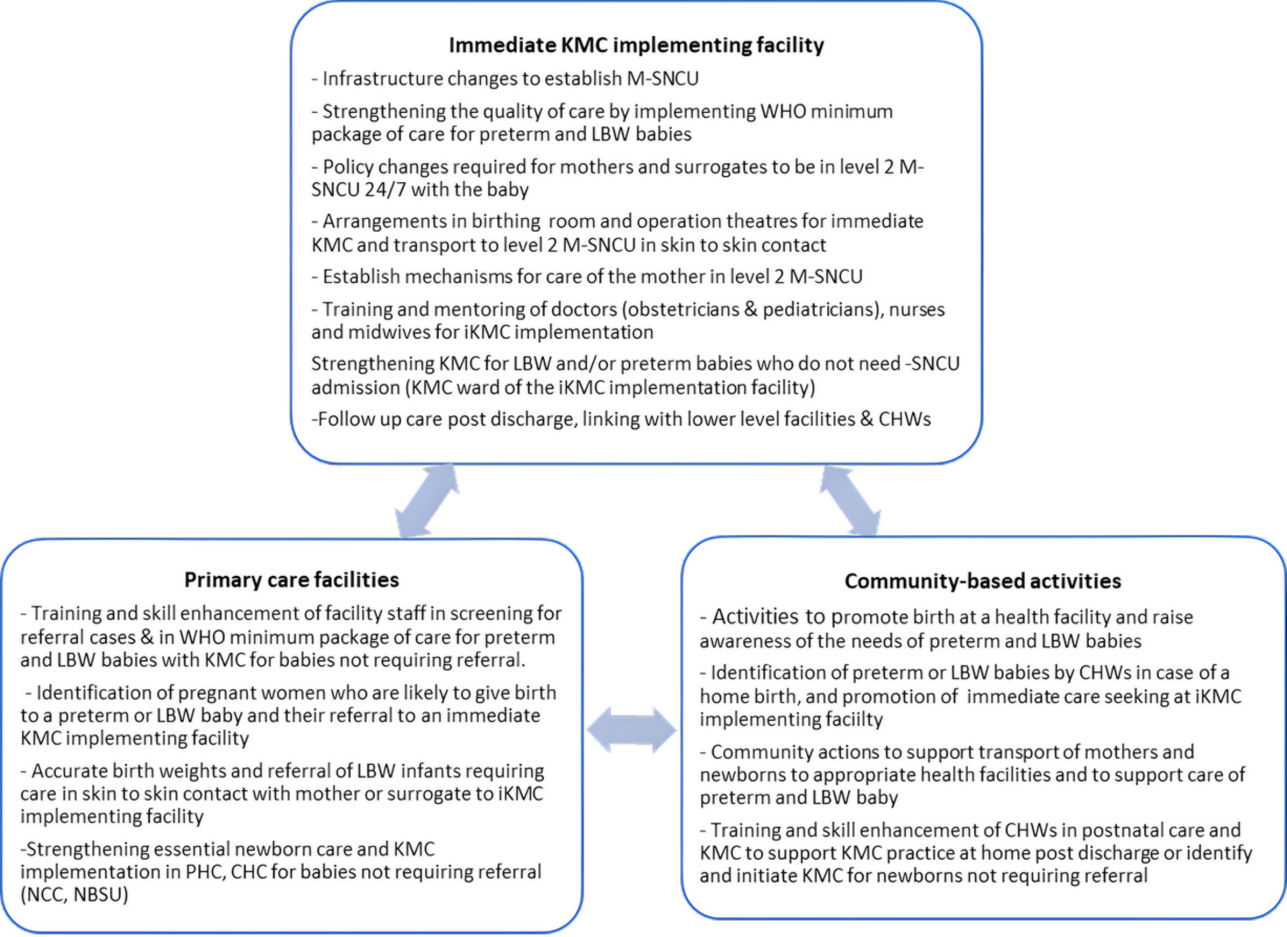
Activities expected in iKMC implementing facilities and communities in model development and optimisation phase.

The above process will be repeated every 3 months. We envisage at least three cycles during model design and optimisation, until reaching a scalable model aiming for high coverage (aspirational for 80% coverage) and quality of iKMC. The WHO team will facilitate the sharing of learnings across different study sites to identify further opportunities to improve the implementation models ( Figures 5 and 6). While the implementation model largely focuses on the hospitals where iKMC will be implemented, additional activities are being conducted in lower-level health facilities and communities in the study districts ( Figure 7).

***3.4.4 Identification of questions related to scalability of the implementation***

Prior to initiation of the scale-up of the model, the research team will engage with the administration, government, and programme managers at national and subnational level to identify the questions of scalability of the model within the existing system. These questions could be related to the scale up pathway and approach, potential barriers, capacity required for scale up, and investments needed. Addressing these questions throughout the duration of the research will help prepare for provincial and national scale-up, which will include development of the scale-up strategy, investment case and implementation roadmap.

***3.4.5 Summary of strategies to develop and optimize an iKMC model***

The first step was the engagement of key stakeholders in the selected study districts. Two concurrent activities were conducted in the first 2-4 months of the project implementation: formative research and strengthening the quality of care by ensuring implementation of the WHO minimum package of care for the small and sick newborn 32.

The formative research team conducted interviews and focus group discussions to explore the domains of the consolidated framework for implementation research as described above. The duration of formative research was relatively short, as this team transitioned into the programme learning team and continues to collect relevant data throughout the model development and optimisation period.

A rapid assessment of newborn care provided in the birthing room, operation theatre and level 2 NICU was conducted to identify gaps in implementation of the WHO minimum package of care for small and sick newborns. Based on the gaps identified, the study supports activities to address these gaps.

When the results of formative research are available, co-design workshops to develop the implementation model 0+ will be organized. This model will be implemented, with ongoing collection of qualitative and quantitative data for 3-6 months, and re-design workshops will revise the model as required. Two or three similar iterative cycles will continue to optimize the implementation model until the end of the model optimisation stage. Phase 1 is completed over a period of 20 months, while phase 2 will be completed in 18 months.

### 3.5 Outcomes for the model development and optimisation phase

***The main outcome*** of this phase will be development of *the optimized implementation model* that achieves high coverage and quality of iKMC and establishes the norm of zero separation between the mother and her infant (in each of the four participating countries).

This will be measured through a primary coverage outcome in a tracer indicator that captures the skin-to-skin component of iKMC i.e., initiation and duration of skin-to-skin contact.

***Primary coverage outcome*:** The proportion of preterm and/or LBW infants born in or brought to the iKMC implementing facility within 24 hours of birth, requiring care in the level 2 M-SNCUs, and received iKMC. This will be captured through a tracer indicator on initiation and duration of skin-to-skin contact.

An infant is considered to have received iKMC if:

• Skin-to-skin contact is initiated within 2 hours after birth if the infant is born within the iKMC implementing facility; if the infant is born outside, then within 2 hours of reaching the iKMC implementing facility (for those infants who reach the facility within 24 hours of birth)
• Continuous skin-to-skin contact is provided by a mother/surrogate for at least 8 hours per day during the stay in the iKMC implementing facility (average hours per day for the overall M-SNCU stay/KMC wards/postnatal wards or elsewhere in iKMC implementing facility)

**Quality outcomes:**

• The proportion of preterm and/or LBW infants who arrived at an iKMC implementing facility within 24 hours after birth among those born in a health facility who required referral at birth to an iKMC implementing facility
• The proportion of preterm and/or LBW infants on respiratory support (any oxygen or CPAP) who received skin-to-skin contact for more than 8 hours per day in the M-SNCU
• The proportion of preterm and/or LBW infants receiving KMC at discharge (8-24 hours of skin-to-skin contact and exclusively breastfed) in the 24 hours before discharge from the iKMC implementing facility
• The proportion of preterm and/or LBW infants who are exclusively breastfed at discharge
• The median age at which the infant is put to the breast for the first time during M-SNCU stay

***Implementation outcomes:***

In the model development and optimisation phase, we will also monitor the following implementation outcomes, including experience of care by mothers/caregivers as part of ongoing programme learning in each site:

• ***Acceptability:*** iKMC, including transport in skin-to-skin contact from the birthing place to M-SNCU, is acceptable to mothers (and/or surrogates). Mothers and caregivers are satisfied with the care received. iKMC is acceptable to health workers in the facility and community.
• ***Adoption:*** The challenges in iKMC adoption by mothers, hospital managers, and health providers (nurses, midwives, doctors) identified in out are addressed.
• ***Adaptation***: Modifications to adapt the iKMC intervention to the social and health system context are implemented.
• ***Fidelity:*** Quality iKMC care is provided to mothers and infants in the level 2- M-SNCUs

Implementation outcomes are not part of the central data management system; however, qualitative information is captured on a regular basis at each site to improve the process and implementation of iKMC as part of programme learning and monitoring ( Table 4).

**Table 4.** Definitions, numerators, and denominators of outcomes in model optimisation phase.

	Numerator and denominator	Specific notes to the indicator	Data collected by	
			Who	How and where
**Primary coverage outcomes**				
Proportion of preterm or LBW newborns requiring care in the M-SNCU born in or brought to the iKMC implementing facility, within 24 hours of birth, who received iKMC.	Numerator: Number of preterm or LBW newborns who received iKMC (in an iKMC implementing facility) Denominator: Number of preterm or LBW newborns requiring care in the M-SNCU born in or brought to the iKMC implementing facility, within 24 hours of birth (Expressed as a percentage)	A baby is considered to have received iKMC if: 1. Skin-to-skin contact is initiated within 2 hours after birth if the baby is born within the iKMC implementing facility, or if the baby is born outside the iKMC implementing facility, within 2 hours of reaching the iKMC implementing facility (who reach the facility within 24 hours of birth) and 2.Skin-to-skin contact is provided by a mother/surrogate for at least 8 hours per day during the stay in the level 2 M-SNCU hospital (average hours per day for the overall M-SNCU stay) and mother’s self-reporting for the past 24 hours)	Outcome measurement team	By checking M-SNCU register/clinical records filled by routine staff in M-SNCU and interviews with mothers at M-SNCU
**Other outcomes**				
Proportion of preterm or LBW infants who arrived to an iKMC implementing facility within 24 hours after birth among those who required referral to an iKMC implementing facility	Numerator: Number of preterm or LBW infants who arrived to an iKMC implementing facility within 24 hours after birth Denominator: Number of preterm or LBW infants* requiring referral to an iKMC implementing facility *Infants requiring referral born in birthing facilities or at home whose birth weight is known		Outcome measurement team	By checking M-SNCU register/clinical records filled by routine staff in M-SNCU At iKMC implementing facility when the baby is admitted Birth weight and or gestational age data will be collected from all birthing facilities for the denominator
Proportion of preterm or LBW infants on respiratory support (any oxygen or CPAP) who received skin-to-skin contact > 8 hours/day in the M-SNCU	Numerator: Newborn who received skin-to-skin contact > 8 hours/day in the M-SNCU Denominator: Preterm or LBW infants on respiratory support in the level 2 M-SNCU (Expressed as a percentage)		Outcome measurement team	By checking M-SNCU register/clinical records filled by routine staff in M-SNCU and interviews with mothers At M-SNCU
Proportion of preterm or LBW infants receiving KMC (8-24 hours of skin-to-skin contact and exclusively breastfed) in the 24 hours before discharge from the iKMC implementing facility	Numerator: Number of preterm or LBW infants receiving 8-24 hours of skin-to-skin contact and exclusively breastfed in the 24 hours before discharge Denominator: Number of preterm or LBW infants who admitted in the M-SNCU in the iKMC implementing facilities who are discharged alive (Expressed as a percentage)		Outcome measurement team	By checking hospital register/clinical records filled by routine staff i ward and interviews with mothers At iKMC implementing facility
Proportion of preterm or LBW infants exclusively breastfed at discharge	Numerator: Infants who are exclusively breastfed Denominator: Preterm or LBW infants requiring M-SNCU care born alive in or brought alive to the iKMC implementing facility i		Outcome measurement team	By checking hospital register/clinical records filled by routine staff i and interviews with mothers at iKMC implementing facility (discharge area)
Median age at putting the baby to breast for the first-time during M-SNCU stay (Expressed in median hours/days)	Median age in hours/days when a preterm or LBW infants is first put to mother’s breast during M-SNCU stay		Outcome measurement team may cross-verify the information using the clinical records.	By registering data on all preterm or LBW infants admitted to M-SNCUs in CRF At iKMC implementing facility when the baby is admitted and interviews with mothers

## 4. Scaling up the optimized iKMC model (Phase 2)

### 4.1 Rationale and policy context

Building on these finding from phase 1, phase 2 is therefore designed to further test the model in its expansion to other geographic zones in three countries (India, Ethiopia and Nigeria), maintaining the strong focus on integrating iKMC into level 2 small and sick newborn care units, ensuring the mother and baby remain physically together and both receive quality care while in the facility, enhancing health system readiness for providing this care, and supporting sustainable national adoption/adaptationin each country.

In the next phase, participating countries will continue in the multi-country initiative, targeting three to four districts or geographic areas in each. At least one iKMC-enabled facility with a Level-2 small and sick newborn care unit will be selected per district or area, embedding the model within existing maternal and newborn health service platforms. The emphasis will be on implementation for scale-up within national health systems, ensuring quality of care throughout the process and generating evidence on impact. Implementation will be accompanied by robust monitoring, evaluation, and adaptive learning mechanisms.

**Country policy contexts**

In Ethiopia, KMC is formally adopted within national newborn health strategies and implementation frameworks, with strong government leadership and partner alignment. The Ministry of Health has endorsed the integration of immediate KMC as part of ongoing efforts to strengthen care for small and sick newborns, supported through major national platforms. National plans now include expansion of KMC to additional facilities across multiple regions, with Sidama region, the iKMC-IR phase 1 district, serving as a learning hub for scale-up. These developments reflect clear policy commitment and coordinated action toward institutionalizing immediate KMC within the national health system.

In India, Mother Newborn Care Unit (MNCU) and immediate KMC are integrated into national newborn care policy, training, and operational frameworks including Facility-Based Newborn Care (FBNC) training document for healthcare providers (in-service doctors and nurses) in 2023. Additionally, a dedicated chapter on MNCU and immediate KMC has been added to paediatric textbooks for both undergraduate and postgraduate students, ensuring the concept is part of the pre-service curriculum. An operational guide for MNCUs has also been developed, outlining unit design, infrastructure costing, human resources, required equipment, and consumables (including KMC binders and garments).

In Nigeria, KMC is included in national newborn health policies and operational guidelines, and its integration within the Nigeria Every Newborn Action Plan reflects strong government commitment to improving survival of preterm and low-birth-weight infants. Over recent years, following the WHO recommendation in 2023, the Federal Ministry of Health has commissioned national newborn committee to develop operational guidance for immediate KMC implementation. Pilot activities in smaller scale in selected facilities are ongoing. This implementation research will lay the foundation for its integration within Level 2 newborn care units. These early experiences mark the starting point for embedding immediate KMC into routine practice and will provide essential learning to inform national scale-up.

### 4.2 Study summary

Phase 2 is a 12-month, multi-country implementation project. The design prioritizes:

• service delivery and health system strengthening
• data collection and performance feedback
• continuous quality improvement cycles
• stakeholder engagement and subnational and national ownership

The project will be implemented concurrently in the selected facilities in 3 – 4 districts per country in the three countries.

***4.2.1 Implementation approach***

This section describes the implementation approach as well as the methods to be used to evaluate the impact of the integration of iKMC on defined outcomes.

The implementation approach is grounded in the principles of health systems strengthening and continuous quality improvement. Implementation will begin concurrently across all selected facilities, ensuring synchronized momentum and shared learning across settings. The iKMC practices will be embedded within the broader framework of strengthened small and sick newborn care emphasizing holistic and integrated service delivery.

To support this integration, targeted capacity-building efforts will be rolled out, including hands-on training, mentoring, and coaching of facility-based teams. Comprehensive monitoring and feedback systems will be established, featuring real-time dashboards and routine data use for adaptive management and quality improvement.

Technical coordination by WHO will provide oversight aimed at ensuring adherence to global standards. This includes quality assurance procedures, adoption of best practices, and supporting continuous institutional learning and sharing of lessons learned within a country and between countries.

After phase 1 ends, we anticipate a three-month preparatory period, during which structural changes, structural changes will occur in selected facilities, staff training will take place, and monitoring systems will be established. In addition, a facility mapping exercise will be conducted to understand existing service delivery, coverage and quality of care in the selected implementing facilities. Information collected will include data on distribution of births, number of preterm and/or low-birthweight (LBW) infants, and referral pathways between the selected implementing facilities (herein referred to as the iKMC implementing facilities) and birthing centres. This will be accomplished through phone interviews with facility managers and analysis of secondary health system data, in place of the comprehensive facility mapping conducted during Phase 1. This reduced exercise is intended to allow for a more manageable process that health facilities can adapt and continue in country efforts to scale-up beyond this project.

Baseline and evaluation data collection activities will concentrate on iKMC implementing facilities. The baseline will be conducted during this three-month preparation period September to November 2025, and its objective will be to measure the outcomes on coverage and quality of care in the iKMC implementing facilities for Phase 2. The baseline data will provide a reference point for evaluation of the impact of scaling-up of the model. Data will be collected monthly until the end of the project and will contribute to the quality improvement efforts. It will also inform the government and key stakeholders of the impact on coverage and quality of care with the scale-up of the implementation model.

A core stakeholder group will be convened at regular intervals, initially to discuss the facility mapping results and consider the qualitative findings from Phase 1 and what is pertinent to their site in Phase 2 and to suggest adaptations to the optimized model for the implementing facilities.

Phase 2 activities can be categorized into two groups: programmatic and evaluation:

Programmatic

I. **System preparation & strengthening (months 1-3)** focuses on preparing health facilities through facility mapping, infrastructure improvements, setting up of the newborn care units/MSNCUs (Mother-special newborn care units described in the original protocol), equipment procurement, staff training, and establishing monitoring systems.
II. **iKMC contextual adaptation and model integration (months 4-12)** entails launching iKMC implementation across all selected iKMC implementing facilities, embedding practices into existing service delivery workflows, rigorous monitoring, and providing continuous mentoring and documentation of lessons learned.

Evaluation

I. Baseline data collection (months 1-3) includes data on coverage and quality of iKMC and the care of the small and sick newborn.
II. **Ongoing evaluation (months 4-9)** entails continued data collection and comprehensive data analysis and feedback.
III. **Final evaluation (months 10-12)** entails continued data collection and comprehensive data analysis and outcome evaluation.

Throughout implementation regular meetings will be held with government and other implementing partners to facilitate the embedding of iKMC and strengthening of small and sick newborn care into the health system and to determine how to scale-up to other geographical areas in the country. A last phase of work will focus on sharing findings through dissemination events at multiple levels of the health system and with global partners.

***4.2.2 Implementation strategies***

This demonstration project will use six main strategies to ensure strong and effective implementation. First, the model will be replicated and adapted to suit new sites and contexts by building directly on the lessons and insights gained during Phase 1. This tailored approach ensures that each site benefits from prior experience while remaining responsive to local needs and conditions.

Second, hands-on mentoring will be delivered by mentors in the countries, guided by the technical support team at WHO, offering practical, on-the-ground support to facility teams. These mentors will play a critical role in capacity building, troubleshooting, and reinforcing adherence to the iKMC model.

Third, rigorous quality assurance mechanisms will be instituted across all participating sites. These will include the use of standardized indicators, regular performance audits, and feedback loops that inform continuous improvement.

Fourth, the project will foster strong stakeholder engagement by actively involving government leaders and health system managers at district, state, and national levels. This engagement is essential for institutional ownership and long-term sustainability.

Fifth, while significant learnings were gained and an implementation model was developed during Phase 1, continuous context-specific adaptive learning will be maintained in Phase 2 to guide implementation, with data informing plan refinement and course corrections. Facilities will be encouraged to identify and address bottlenecks proactively. However, in this phase, the timeline is more compressed, requiring more streamlined data collection, quicker decision-making and faster implementation of solutions.

Lastly, WHO will provide overarching technical oversight and coordination; implementation will benefit from established standard data management systems for quality monitoring, application of global best practices, compliance with health standards, and systematic sharing of cross-country learnings.

***4.2.3 Evaluation design***

Outcomes for the model scale up phase

All outcomes will be measured in the population enrolled in the project (as defined above). Table 5 below provides further detail on each outcome.

**Table 5.** Definitions, numerators, and denominators of outcomes.

	Numerator and denominator	Specific notes to the indicator	Data collected by	
			Who	How and where
**Primary coverage outcome**				
iKMC coverage for inborn babies	Numerator: Number of preterm and/or LBW newborns requiring care in level 2 newborn care unit/M-SNCU born in the iKMC implementing facility who received iKMC Denominator: Number of preterm and/or LBW newborns requiring care in level 2 newborn care unit/M-SNCU born in the iKMC implementing facility (Expressed as a percentage)	The tracer indicator is on initiation and duration of skin-to-skin contact A baby is considered to have received iKMC if: 1. Skin-to-skin contact is initiated within 2 hours after birth AND 2.Skin-to-skin contact is provided by a mother/surrogate for ≥ 8 hours per day during the stay in the iKMC implementing facility (average hours per day for the overall stay)	Outcome measurement team	By checking registers, KMC monitoring sheet, interviewing mothers (method established in Phase 1 at each country/site)
iKMC coverage for outborn babies	Numerator: Number of outborn preterm and/or LBW newborns requiring care in level 2 newborn care unit/M-SNCU brought to the iKMC implementing facility within 24 hours of birth who received iKMC in the iKMC implementing facility Denominator: Number of preterm and/or LBW newborns requiring care in level 2 newborn care unit/M-SNCU brought to the iKMC implementing facility within 24 hours of birth (Expressed as a percentage)	A baby is considered to have received iKMC if: 1. Skin-to-skin contact is initiated within 2 hours of reaching the iKMC implementing facility within 24 hours of birth AND 2.Skin-to-skin contact is provided by a mother/surrogate for ≥ 8 hours per day during the stay in the iKMC implementing facility (average hours per day for the overall stay)	Outcome measurement team	By checking registers, KMC monitoring sheet, interviewing mothers (method established in Phase 1 at each country/site)
**Quality outcomes**				
Respiratory support and skin-to-skin contact	Numerator: Number of preterm and/or LBW infants on respiratory support in the level 2 newborn care unit/MSNCU in the iKMC implementing facility who received skin-to-skin contact ≥8 hours/day Denominator: Preterm or LBW infants on respiratory support in the level 2 newborn care unit/MSNCU in the iKMC implementing facility (Expressed as a percentage)		Outcome measurement team	By checking registers, clinical records, KMC monitoring sheet, interviewing mothers (method established in Phase 1 at each country/site)
Early breastfeeding initiation	Median age in hours/days when an enrolled preterm and/or LBW infant is first put to the mother’s breast		Outcome measurement team may cross-verify the information using the clinical records.	By checking registers, KMC monitoring sheet, interviewing mothers (method established in Phase 1 at each country/site)
Early breast milk feeding initiation	Median age in hours/days when an enrolled preterm and/or LBW infant receives first feeding with human milk (either directly from the breast or indirectly expressed breast milk)		Outcome measurement team may cross-verify the information using the clinical records.	By checking registers, KMC monitoring sheet, interviewing mothers (method established in Phase 1 at each country/site)
Effective KMC at discharge	Numerator: Number of enrolled preterm and/or LBW infants who are receiving skin-to-skin care ≥ 8 hours AND exclusively breast milk fed for the previous 24 hours at the time of discharge from the iKMC implementing facility Denominator: Enrolled preterm and/or LBW infants alive at discharge		Outcome measurement team	By checking registers, clinical records, KMC monitoring sheet, interviewing mothers (discharge area) (method established in Phase 1 at each country/site)

Primary coverage outcome

1. iKMC coverage for inborn babies: proportion of preterm or LBW newborns born in the iKMC implementing facility, who received iKMC.
2. iKMC coverage for outborn babies: proportion of preterm or LBW newborns brought to the iKMC implementing facility within 24 hours of birth, who received iKMC.

These will be captured through tracer indicators on initiation and duration of skin-to-skin contact.

Quality outcomes

1. Respiratory support and skin-to-skin contact: proportion of preterm and/or LBW infants on respiratory support (any oxygen or CPAP) in the newborn care unit/MSNCU who received skin-to-skin contact ≥ 8 hours/day
2. Early breastfeeding initiation: median age at putting the baby to breast for the first time (Expressed in median hours/days)
3. Early breast milk feeding initiation: median age of first feeding with human milk (Expressed in median hours/days)
4. Effective KMC at discharge: proportion of preterm and/or LBW infants receiving KMC (skin-to-skin care ≥ 8 hours and exclusively breast milk fed) at discharge from the iKMC implementing facility

## 5. Cost

Evaluation of costs of the optimized implementation model is essential to provide economic evidence and financial implications for policymakers to adopt the model. Specifically, the following information will be collected: Estimates of the total additional annualized cost of including the model into the country level health systems, including cost per bed and child to receive intervention in the M-SNCU.

Costs will firstly be categorized as: fixed costs that would include capital costs related to infrastructure (including construction or renovation costs), costs of buying equipment for the M-SNCU, and training costs. Recurrent costs will include costs related to the regular functioning of the M-SNCU. We will use standard methods in consultation with health economics experts.

## 6. Recruitment of participants, data collection and data management

### 6.1 Recruitment of participants

Participants for formative research will be identified for the different activities described above in Section 3.1. Maximum variation sampling will be used to encourage recruitment and sampling to reach research participants with diverse characteristics. For example, women who had preterm and/or LBW infants to be interviewed should include women of different ages, geographic zones, parity, and ethnicities. As data will be analyzed in parallel with data collection, we will look for thematic data saturation and adjust the sample size as necessary (e.g., discontinuing further interviews if saturation is deemed achieved, or conducting additional interviews as needed until saturation is deemed achieved). The research teams will facilitate contact with potentially eligible participants. Each individual will be provided with an information sheet about the study, invited to participate by the research team, and if they agree, asked to provide consent.

At the iKMC implementing facility, research assistants will identify eligible mothers and infants from facility registers in the labour room and operation theatre for those born in the facility; or in the emergency, OPD, SNCU register for those infants who were referred to iKMC implementing facility. A register will be created and will have names of all preterm or LBW infants in all facilities. The research assistant will have access to this register to ensure that all eligible mothers and infants are enrolled in the study.

### 6.2 Data collection

***6.2.1 Formative research and programme learning***

Information on the different data collection activities during the formative research is outlined above in Section 3.1. Instruments, or semi-structured discussion guides, for the different activities will be developed guided by barriers and facilitators learned from the iKMC trial and the scale-up KMC study. We will also explore the feasibility of proposed activities. All instruments will be reviewed and adapted by the study teams in each site and translated and tested prior to data collection, as part of training of the research teams. Draft instruments are submitted separately.

Data will be analysed in parallel with data collection, monitoring for thematic data saturation. Interviews and discussions will be digitally recorded, and notes of each session will be taken, including descriptive and reflective information. All notes will be anonymized to ensure privacy and confidentiality of participants. Participants and sites will be given identifying numbers for reference. All data will be stored and backed up on secured password- protected system and will not be accessed nor shared with a third party without prior permission.

The team will identify key themes emerging from the notes of the different activities and will report extracts to provide examples of specific issues. Themes identified will be mapped to the different domains of the Consolidated Framework for Implementation Research and to key areas related to iKMC intervention implementation, identifying key barriers and facilitators for iKMC implementation.

***6.2.2 Model optimisation***

The evaluation team will collect birth weight data of live births in all health facilities in the district through periodic visits to these facilities. The number of preterm and/or LBW infants requiring admission to the level 2 M-SNCU will be documented based on the admission criteria for that unit (e.g., <1.8 kg). They will also record the date and time of birth for such LBW infants born in iKMC implementing facilities, as well as date and time of birth and of admission for infants born at lower-level facilities and at home.

The outcome measurement team will collect the data on the time of initiation of iKMC and who initiated iKMC through daily visits to newborn care units in iKMC implementing facilities in the district. For infants born at home or in a primary level facility and brought to iKMC implementing facility, information on the time of initiation of KMC will be ascertained from the mother or another caregiver and data will also be taken from the register in the primary level facility. Daily information on the hours of skin-to-skin contact will be collected by the evaluation team (by asking the mothers) while the infant is in the iKMC implementing facility. Staff at the iKMC implementing facilities will record the time of initiation and duration of KMC in hospital records.

***6.2.3 Optimised model scale-up***

For the optimized model scale-up phase, research assistants will identify eligible mothers and babies from facility registers in the labour room and operation theatre for those born in the iKMC implementing facility; or in the emergency, OPD, SNCU register for those babies who were referred to iKMC implementing facility. A register will be created and will have names of all preterm or LBW babies in all facilities. The research assistant will have access to this register to ensure that all eligible mothers and babies are enrolled in the study.

The Study team will train and supervise staff at the iKMC implementing facilities to record above mentioned information in the register accurately. Spot checks will be conducted by the study team to ensure quality of data collected in the iKMC implementing facilities. Ongoing monitoring and discussions by the WHO team will also include regular review of data.

### 6.4 Data management

In partnership with local research institutions, a dedicated common data management platform will be developed in a bespoke database system with a systems-based MS SQL server and the front-end interface developed in Dot NET. Process and outcome data will be captured using the android devices in offline mode. This data will be synchronized to the site server daily and fortnightly into the central server.

To ensure the data quality and data integrity, data quality rules (logical, range and consistency checks) will be incorporated into the system. A two-way data synchronization will be setup in the data management system to ensure that the site server and the data collector’s machine have been updated to implement the data integrity checks. A data monitoring module (dashboard) will have features such as to produce data outputs for regular data quality check, generation of the visit schedules and data completeness checks and analysable data set.

Each team will be responsible for data management for its site. Each site will have a data manager who will be responsible for performing data quality checks, managing local query, and ensuring correctness and completeness of information in CRFs. Query management will be performed online.

A comprehensive data management plan describing the data management activities from CVT finalization, database development, data entry procedure, data handling [including edit checks, range checks and query management], risk management, database lock and transfer will be developed to organise and manage the data ensuring data integrity and security throughout the study duration. Central data management team will share the query management reports with the site on a fortnightly/monthly basis to resolve the queries within the defined period and to update the data, if required.

### 6.5 Confidentiality

The following measures will be taken to ensure participant confidentiality:

• Study data for each participant will be identified by a unique anonymous ID number.
• The local study register linking personal information and trial ID numbers, and all personal information of participants, will be kept separate from the CRFs
• Study documents will be kept securely under lock and key in the research offices and will not be accessible, other than to the researchers
• Data will be entered by study ID number in the password-protected data management system to which only study staff will have access
• The study report will not contain the names of any participants
• After completion of the study all study documents, including audio recordings, will be archived in accordance with institutional and national rules for clinical research archiving for a minimum period of 10 years.
• The data will be digitally archived for permanent storage.

A good clinical practice -compliant data management system will be used to protect data in every aspect of data management from data collection to data analysis. Data will be linked to participant identification numbers and each site will have a data manager responsible for ensuring data quality. To facilitate harmonization of data and ensure data quality, we will use a standard set of case report forms (CRFs); core variable tables; and data collection processes. Data will be password protected and stored in a local server of each site. Once entered, data will be synchronized to a central server every two weeks and locked immediately after all queries are resolved. An automated database backup module will be used to take the database backup daily. One copy of the database will be stored in an encrypted form in another computer in the network and one copy will be maintained outside the office premise under the strict supervision of the data manager.

Data quality improvement: Imbedded range and consistency checks in electronic CRF (online real-time checks) will be put in place at the time of data collection. A scheduled, two-layer data quality check for completeness and accuracy will be implemented both at the site and at the WHO Data Coordination Centre. Data is owned by each study site. The study site will share cleaned de-identified cumulative dataset (with participant ID) with WHO for review and feedback through secure password protected systems. Monthly data monitoring reports will be shared with study sites and WHO study coordination team to monitor progress and quality of data. Random spot checks and data validation will be coupled with independent audits.

## 7. Ethical considerations

All project activities will be conducted in accordance with international norms and standards on ethical research practice, including the Declaration of Helsinki and the Council for International Organizations of Medical Sciences International Ethical Guidelines for Health-related Research Involving Humans. ^49,50,51^

The Research Ethics Review Committee of the World Health Organization (WHO ERC) has reviewed and approved the study protocol (Protocol ID: ERC.0003880). In addition, the institutional review boards associated with each participating study site have reviewed and approved the protocol in accordance with their local regulations.

Prior to the start of the study at each site, the principal investigator (PI) and implementation research team will conduct orientation visits to the participating health facilities. During these visits, health facility administrators, clinical staff, and - where applicable - community representatives will be informed about the study objectives, procedures, and data collection methods. Country leads will facilitate any study-related monitoring, regulatory inspections, audits, and ethical review at their site as required.

Current evidence showed the study intervention itself has proven to be safe and efficacious. As per the recently released WHO recommendations, ^4^ provision of KMC immediate after birth to all preterm or LBW babies will be considered the standard of care); therefore, initiation of iKMC will not require consent from participants. Nonetheless, a blanket permission will be obtained from the facility manager to allow the research team access to facility registers in order to identify eligible mothers and babies based on birth weight, gestational age and status of SNCU admission. In addition, information will be posted in the facility to inform women and families about the research. Facility staff will be briefed about the process to follow if someone requests an opt out. A written informed consent will be obtained by the research assistant for all activities during formative research and programme learning. A written informed consent will also be obtained from the mother by the research assistant for outcome measurements and programme learning that includes direct data collection from mothers and clinical records in both phases of the study, upon admission to the SNCU/M-SNCU. Included in the consent form for the stepped wedge trial phase is consent to follow up on day 29 to determine the vital status of the baby. A verbal confirmation to verify the mother’s consent for follow up and her contact information will be obtained by the research assistant at discharge.

Mothers who are minors will be eligible for enrolment in this study. Pregnant minors are considered emancipated minors in Nigeria and Ethiopia; in this case co-consent from their parent or guardian is not required. In India and Bangladesh, a written assent from the minor mother will first be obtained and co-consent form will be signed by the minor mother’s guardian (parent). A legally acceptable representative can be either of the parents or the guardian of infant in the absence of parents. When explaining the details in the information sheet, the study team member will ask if minor mothers or the parent or guardian has any questions and if there are any, will answer the questions.

In the case of disagreement between a minor and the parent or if the parents are not available, we will follow local study procedure in each site, based on national legal frameworks, site experience and local culture. All consent forms, including assent forms, in the study will be translated into a local language that facilitates the consent process. For illiterate participants the consent form will be read by the research staff in presence of a literate witness.

Additionally checking questions will be asked to assess the participants’ understanding about the study. Once the team is satisfied that the study procedures are well understood, consent will be documented. A copy of the signed consent form will be returned to the participants.

In the consent process, participants will be briefed that the mother and baby will continue to receive standard care as per hospital policy in case of refusal to participate in the study or withdrawal of consent. Mothers will be made aware that even after providing consent, they will be free to withdraw from the study at any stage and in case they withdraw later, they and their babies will continue to receive the same quality of care as required by any other patient with the same condition. At the time of withdrawal of consent, the mothers will be free to decide about use of collected data.

## 8. Dissemination

The study team plans to disseminate findings among global, national and sub-national stakeholders through in-country dissemination events and globally through peer-reviewed journal manuscripts and international conference presentations.

## 9. Discussions

This study represents the first implementation research focused on understanding how to integrate iKMC into care for small and sick infants into the existing health systems effectively. With iKMC now a global recommendation, many countries, particularly those with limited resources, may face challenges in its practical application. This research offers valuable programmatic learnings into overcoming these challenges and identifies opportunities for scaling up iKMC, not only for the broader national context within the study countries but also for other countries considering implementing iKMC in strengthening small sick newborn care in future. These insights offer guidance for policymakers, healthcare providers, and programme implementers, contributing to the effective integration of iKMC into routine newborn care.

A key strength of the study is its use of theoretical frameworks such as APEASE that provide structured guidance for model optimisation through programme learning. Additionally, its global representation across Africa and Asia enhances the generalizability of the findings, making them relevant to diverse contexts. With the recognition of the burden of newborn mortality and the demonstrated interest of governments in all four countries to ensure availability of iKMC for all small and sick newborns, the timing is opportune for implementing this study. It provides a great opportunity to integrate iKMC into existing health systems and, through government collaboration, identify scalable and sustainable implementation models. This study design enables ongoing learning and adaptation, refining the iKMC implementation model and bolstering broader national initiatives. For example, the study improves data reporting by capturing and analysing relevant key indicators related to KMC practices, coverage, and processes, which will eventually improve health management information systems in countries. By generating evidence on how to effectively implement iKMC in diverse settings, the research supports ENAP’s objective to scale up evidence-based interventions for reducing newborn mortality.

## Rights and permissions

The Corresponding Author has the right to grant on behalf of all authors and does grant on behalf of all authors, a worldwide license to the Publishers and its licensees in perpetuity, in all forms, formats and media (whether known now or created in the future), to i) publish, reproduce, distribute, display and store the Contribution, ii) translate the Contribution into other languages, create adaptations, reprints, include within collections and create summaries, extracts and/or, abstracts of the Contribution, iii) create any other derivative work(s) based on the Contribution, iv) to exploit all subsidiary rights in the Contribution, v) the inclusion of electronic links from the Contribution to third party material where-ever it may be located; and, vi) license any third party to do any or all of the above.

## Data Availability

The datasets generated during the current study are available from WHO on request in future. Open Science Framework: Integrating immediate Kangaroo Mother Care into district hospitals with a level 2 neonatal intensive care unit: Implementation Research Protocol Annexes, https://doi.org/10.17605/OSF.IO/G4P7B. ^19^ Data are available under the terms of the Creative Commons Attribution 4.0 International License (CC BY 4.0)
